# Supplementary material for: Rationale and Design of the Future Optimal Research and Care Evaluation in Patients with Acute Coronary Syndrome (FORCE-ACS) Registry: Towards “Personalized Medicine” in Daily Clinical Practice
Source: J Clin Med. 2020 Sep 30;9(10):3173. doi: 10.3390/jcm9103173 (PMC7601438; doi:10.3390/jcm9103173)
Supplement: Supplementary file 1 [file jcm-09-03173-s001.pdf]

## Supplementary Appendix – FORCE-ACS Registry

## Variable definitions

**Cardiovascular risk factors:**

- Smoking[1]
  Differentiation between non-smoker, current smoker or stopped smoker
    - o Current smoker if active smoking in the past 4 weeks
    - o Stopped smoker if stopped >4 weeks ago

- Hypertension (HT)[1]:
  Differentiation between treated or untreated HT
    - o Systolic ≥140 mmHg and/or diastolic ≥90 mmHg in at least 2 separate measurements OR
    - o HT in medical history OR
    - o Current use of antihypertensive medicine(s)

- Hypercholesterolemia (HC)[1]:
  Differentiation between treated or untreated HC
    - o Total cholesterol ≥ 5.0 mmol/l and/or LDL-cholesterol ≥2.5 mmol/l OR
    - o HC in medical history OR
    - o Current use of lipid lowering medicine(s)

- Diabetes Mellitus (DM):
    - o DM Type I, II or undifferentiated/other/unknown type in medical history

- Positive family history for cardiovascular disease (CVD):
    - o Known CVD (coronary artery disease or MI, stroke, peripheral artery disease or other forms of atherosclerosis) in first-degree relatives (parents, siblings, offspring) <60 years

*(1) 2016 ESC Guidelines on cardiovascular disease prevention in clinical practice. European Heart Journal (2016) 37, 2315–2381. doi:10.1093/eurheartj/ehw106*

**Medical history:**

- Myocardial infarction (MI):
    - o Medical history notes a myocardial infarction, according to 'Fourth universal definitions of myocardial injury and myocardial infarction', ESC 2019 (See endpoints)

- Percutaneous coronary intervention (PCI) with stent placement
    - o Medical history notes a PCI with coronary stent placement

    If present:
    - Previous stent thrombosis
        - o Medical history notes a stent thrombosis

- Coronary artery bypass grafting (CABG)
    - o Medical history notes coronary artery bypass grafting

- Previous clinically relevant bleeding
    - o Clinically relevant: any bleeding in medical history for which medical assessment was needed

  Differentiate between spontaneous and non-spontaneous:

- Spontaneous (non-intervention related or non-traumatic bleeding)
- Non spontaneous (intervention related or due to traumatic cause)

- Stroke
  - Medical history notes a stroke

  Differentiate between ischemic, hemorrhagic and/or undifferentiated stroke

  Differentiate between cerebral vascular accident (CVA) and/or transient ischemic attack (TIA):
    - CVA will be defined as an acute new neurological deficit ending in death or lasting >24 hours not due to another readily identifiable cause such as trauma.
    - TIA will be defined as an acute new neurological deficit lasting < 24 hours not due to another readily identifiable cause such as trauma

- Peripheral artery disease (PAD)

  Medical history notes proven PAD:

  Definition used in the COMPASS trial: (*Eikelboom et al. NEJM 2017 DOI: 10.1056/NEJMoa1709118)*
  - Previous aorto-femoral bypass surgery, limb bypass surgery, or percutaneous transluminal angioplasty revascularization of the iliac, or infra-inguinal arteries, OR
  - Previous limb or foot amputation for arterial vascular disease, OR
  - History of intermittent claudication and one or more of the following:

    1) An ankle/arm blood pressure (BP) ratio < 0.90, OR

    2) Significant peripheral artery stenosis (≥50%) documented by angiography, or by duplex ultrasound, OR
  - Previous carotid revascularization or asymptomatic carotid artery stenosis ≥50% as diagnosed by duplex ultrasound or angiography.

- Heart failure
  - Medical history notes heart failure

- Left ventricular ejection fraction (LVEF)
  - Differentiate between =>50%, 30-50%, <30% or unknown

- Chronic renal insufficiency, defined as
  - The presence of GFR <60 mL/min/1.73 m2 for 3 months, with or without other signs of kidney damage.

  If present:
  - Dialysis, defined as
    - Renal failure with dialysis treatment

- Active malignancy
  - Medical history notes active malignancy (i.e. malignancy, excluding non-melanoma skin cancers, with treatment in the last 12 months)

- COPD
  - Medical history notes chronic obstructive pulmonary disease

- Atrial fibrillation
  - Medical history notes atrial fibrillation

**Clinical variables:**

Clinical variables (e.g. height, weight, blood pressure, pulse, etc.) and laboratory values (e.g. hemoglobin, thrombocytes, leukocytes, creatinine, C-reactive protein, etc.) at hospital admission are taken into account. Differences in (laboratory) measurements between participating hospitals (e.g. type of assay used for cardiac troponin, standard units for cardiac troponin, etc.) will be documented. Relevant variables on invasive diagnostics and treatment during admission and at discharge (coronary angiography, PCI, CABG and medical therapy) will also be documented.

**Study end point definitions:**

## Mortality

***ARC-2 criteria***

*Garcia-Garcia et al. Standardized End Point Definitions for Coronary Intervention Trials. The Academic Research Consortium-2 Consensus Document Circulation. 2018;137:2635–2650. DOI:10.1161/CIRCULATIONAHA.117.029289*

| ***Type of Death*** | ***Definition*** |
|---|---|
| *Cardiovascular death* | *Cardiovascular death is defined as death resulting from cardiovascular causes. The following categories may be collected:*<br>*1. Death caused by acute MI*<br>*2. Death caused by sudden cardiac, including unwitnessed, death*<br>*3. Death resulting from heart failure*<br>*4. Death caused by stroke*<br>*5. Death caused by cardiovascular procedures*<br>*6. Death resulting from cardiovascular hemorrhage*<br>*7. Death resulting from other cardiovascular cause* |
| *Noncardiovascular death* | *Noncardiovascular death is defined as any death that is not thought to be the result of a cardiovascular cause. The following categories may be collected:*<br>*1. Death resulting from malignancy*<br>*2. Death resulting from pulmonary causes*<br>*3. Death caused by infection (includes sepsis)*<br>*4. Death resulting from gastrointestinal causes*<br>*5. Death resulting from accident/trauma*<br>*6. Death caused by other noncardiovascular organ failure*<br>*7. Death resulting from other noncardiovascular cause* |
| *Undetermined* | *Undetermined cause of death is defined as a death not attributable to any other category because of the absence of any relevant source documents. Such deaths will be classified as cardiovascular for end point determination.* |

# Myocardial infarction (MI)

***Fourth universal definitions of myocardial injury and myocardial infarction***

*Thygesen et al. European Heart Journal (2018) 00, 1–33. DOI:10.1093/eurheartj/ehy462*

***Criteria for myocardial injury***

*The term myocardial injury should be used when there is evidence of elevated cardiac troponin values (cTn) with at least one value above the 99th percentile upper reference limit (URL). The myocardial injury is considered acute if there is a rise and/or fall of cTn values.*

***Criteria for acute myocardial infarction (types 1, 2 and 3 MI)***

*The term acute myocardial infarction should be used when there is acute myocardial injury with clinical evidence of acute myocardial ischaemia and with detection of a rise and/or fall of cTn values with at least one value above the 99th percentile URL and at least one of the following:*

- *Symptoms of myocardial ischaemia;*
- *New ischaemic ECG changes;*
- *Development of pathological Q waves;*
- *Imaging evidence of new loss of viable myocardium or new regional wall motion abnormality in a pattern consistent with an ischaemic aetiology;*
- *Identification of a coronary thrombus by angiography or autopsy (not for types 2 or 3 MIs).*

*- Post-mortem demonstration of acute athero-thrombosis in the artery supplying the infarcted myocardium meets criteria for type 1 MI.*
*- Evidence of an imbalance between myocardial oxygen supply and demand unrelated to acute athero-thrombosis meets criteria for type 2 MI.*
*- Cardiac death in patients with symptoms suggestive of myocardial ischaemia and presumed new ischaemic ECG changes before cTn values become available or abnormal meets criteria for type 3 MI.*

***Criteria for coronary procedure-related myocardial infarction (types 4 and 5 MI)***

*- Percutaneous coronary intervention (PCI) related MI is termed type 4a MI.*
*- Coronary artery bypass grafting (CABG) related MI is termed type 5 MI.*
*- Coronary procedure-related MI ≤ 48 hours after the index procedure is arbitrarily defined by an elevation of cTn values > 5 times for type 4a MI and > 10 times for type 5 MI of the 99th percentile URL in patients with normal baseline values. Patients with elevated pre-procedural cTn values, in whom the pre-procedural cTn level are stable (≤ 20% variation) or falling, must meet the criteria fora > 5 or > 10 fold increase and manifest a change from the baseline value of > 20%. In addition with at least one of the following:*

- *New ischaemic ECG changes (this criterion is related to type 4a MI only);*
- *Development of new pathological Q waves;*

- *Imaging evidence of loss of viable myocardium that is presumed to be new and in a pattern consistent with an ischaemic aetiology;*
- *Angiographic findings consistent with a procedural flow-limiting complication such as coronary dissection, occlusion of a major epicardial artery or graft, side-branch occlusion-thrombus, disruption of collateral flow or distal embolization.*

*- Isolated development of new pathological Q waves meets the type 4a MI or type 5 MI criteria with either revascularization procedure if cTn values are elevated and rising but less than the pre-specified thresholds for PCI and CABG.*

*- Other types of 4 MI include type 4b MI stent thrombosis and type 4c MI restenosis that both meet type 1 MI criteria. Post-mortem demonstration of a procedure-related thrombus meets the type 4a MI criteria or type 4b MI criteria if associated with a stent.*

***Criteria for prior or silent/unrecognized myocardial infarction***

*Any one of the following criteria meets the diagnosis for prior or silent/unrecognized MI:*

- *Abnormal Q waves with or without symptoms in the absence of non-ischaemic causes.*
- *Imaging evidence of loss of viable myocardium in a pattern consistent with ischaemic aetiology.*
- *Patho-anatomical findings of a prior MI.*

***<u>Differentiation between will be made between STEMI, NSTE-ACS (NSTEMI and UA):</u>***

- *ST-elevation myocardial infarction (STEMI): Patients with acute chest pain, persistent (>20 min) ST-segment elevation and signs of myocardial infarction, as defined by the Third universal definition of myocardial infarction (ESC Guidelines 2012).*
- *Non ST elevation – acute coronary syndrome (NSTE-ACS): Patients with acute chest pain but no persistent ST-segment elevation. ECG changes may include transient ST-segment elevation, persistent or transient ST-segment depression, T-wave inversion, flat T waves or pseudo-normalization of T waves or the ECG may be normal. NSTE-ACS can be further classified into:*
    - *Non ST elevation – myocardial infarction (NSTEMI): Myocardial infarction without the presence of ST- elevation on the ECG, defined by the third universal definition of myocardial infarction (ESC Guidelines 2012)*
    - *Unstable angina (UA): Defined as myocardial ischaemia at rest or minimal exertion in the absence of cardiomyocyte necrosis.*

***<u>Differentiation between type 1 and type 2 myocardial infarction will be made:</u>***

- *Type 1 MI (often STEMI or NSTEMI) is characterized by atherosclerotic plaque rupture, ulceration, fissure, erosion or dissection with resulting intraluminal thrombus in one or more coronary arteries leading to decreased myocardial blood flow and/or distal embolization and subsequent myocardial necrosis). The patient may have underlying severe coronary artery disease (CAD), but, on occasion*

*there may be non-obstructive coronary atherosclerosis or no angiographic evidence of CAD, particularly in women.*

- *Type 2 MI is myocardial necrosis in which a condition other than coronary plaque instability contributes to an imbalance between myocardial oxygen supply and demand. Mechanisms include coronary artery spasm, coronary endothelial dysfunction, tachyarrhythmias, bradyarrhythmias, anaemia, respiratory failure, hypotension and severe hypertension.*
  *In addition, in critically ill patients and in patients undergoing major non-cardiac surgery, myocardial necrosis may be related to injurious effects of pharmacological agents and toxins.*

*2015 ESC Guidelines for the management of acute coronary syndromes in patients presenting without persistent ST-segment elevation. (European Heart Journal (2016) 37, 267–315, doi:10.1093/eurheartj/ehv320)*

## Stent thrombosis

***ARC-2 criteria***
*Garcia-Garcia et al. Standardized End Point Definitions for Coronary Intervention Trials. The Academic Research Consortium-2 Consensus Document Circulation. 2018;137:2635–2650. DOI:10.1161/CIRCULATIONAHA.117.029289*

| ***Classification*** | ***Criteria*** |
| --- | --- |
| *Definite stent/scaffold thrombosis* | *Angiographic confirmation of stent/scaffold thrombosis** <br> *The presence of a thrombus† that originates in the stent/scaffold or in the segment 5 mm proximal or distal to the stent/scaffold or in a side branch originating from the stented/scaffolded segment and the presence of at least 1 of the following criteria:* <br> *- Acute onset of ischemic symptoms at rest* <br> *- New electrocardiographic changes suggestive of acute ischemia* <br> *- Typical rise and fall in cardiac biomarkers (refer to definition of spontaneous myocardial infarction)* <br> *Or* <br> *Pathological confirmation of stent/scaffold thrombosis:* <br> *- Evidence of recent thrombus within the stent/scaffold determined at autopsy* <br> *- Examination of tissue retrieved following thrombectomy (visual/histology)* |
| *Probable stent/scaffold thrombosis* | *Regardless of the time after the index procedure, any myocardial infarction that is related to documented acute ischemia in the territory of the implanted stent/scaffold without angiographic confirmation of stent/scaffold thrombosis and in the absence of any other obvious cause.‡* |

| | |
|---|---|
| *Silent stent/scaffold occlusion* | *The incidental angiographic documentation of stent occlusion in the absence of clinical signs or symptoms is not considered stent thrombosis.* |

***Timing of ST (duration after stent implantation)***

| | |
|---|---|
| *Acute* | *0§–24 h* |
| *Subacute* | *>24 h–30 d* |
| *Late* | *>30 d–1 y* |
| *Very late* | *>1 y* |

***NB:***

*- Early stent thrombosis is 0 to 30 days (acute plus subacute stent thrombosis).*
**Definite stent/scaffold thrombosis is considered to have occurred by either angiographic or pathological confirmation.*
*†Occlusive thrombus: Thrombolysis in Myocardial Infarction grade 0 or 1 flow within or proximal to a stent/scaffold segment. Nonocclusive thrombus: intracoronary thrombus is defined as a (spherical, ovoid, or irregular) noncalcified filling defect or lucency surrounded by contrast material (on 3 sides or within a coronary stenosis) seen in multiple projections, persistence of contrast material within the lumen, or visible embolization of intraluminal material downstream.*
*‡When the stented/scaffolded segment is in the left circumflex coronary artery or in the presence of preexisting electrocardiographic abnormalities (eg, left bundle branch block, paced rhythms), definitive evidence of localization may be absent and Clinical Events Committee adjudication is based on review of all available evidence).*
*§Defined as the moment the patient is undraped and taken off the catheterization table.*

## <u>Revascularization</u>

***ARC-2 criteria***

*Garcia-Garcia et al. Standardized End Point Definitions for Coronary Intervention Trials. The Academic Research Consortium-2 Consensus Document Circulation. 2018;137:2635–2650. DOI:10.1161/CIRCULATIONAHA.117.029289*

*During follow-up, all percutaneous coronary interventions (PCI) and/or coronary artery bypass grafts (CABG) will be recorded. Differentiation will be made between urgent and non-urgent procedures.*

*Further differentiation will be made between:*

- *PCI with stenting*
- *PCI without stenting (plain old balloon angioplasty, POBA),*
- *CABG.*

*Also, differentiation will be made between:*

- *non-target vessel revascularization (non-TVR)*

- *target vessel revascularization (TVR), further divided into*
    - *target lesion revascularization (TLR)*
    - *non-TLR.*

*Definitions of TLR and TVR, according to the ARC-2 criteria (Garcia-Garcia et al. Circulation 2018):*

- *TLR is defined as any repeat percutaneous intervention of the target lesion or bypass surgery of the target vessel performed for restenosis or other complication of the target lesion. The target lesion is defined as the treated segment from 5 mm proximal to the stent and to 5 mm distal to the stent.*
- *TVR is defined as any repeat percutaneous intervention or surgical bypass of any segment of the target vessel. The target vessel is defined as the entire major coronary vessel proximal and distal to the target lesion, which includes upstream and downstream branches and the target lesion itself.*

## Stroke

***VARC criteria.***

*Kappetein et al. Eur Heart J 2012 Oct;33(19):2403-18. doi: 10.1093/eurheartj/ehs255.*

*Diagnostic criteria*

I. *Acute episode of a focal or global neurological deficit with at least one of the following: change in the level of consciousness, hemiplegia, hemiparesis, numbness, or sensory loss affecting one side of the body, dysphasia or aphasia, hemianopia, amaurosis fugax, or other neurological signs or symptoms consistent with stroke*
II. *Stroke: duration of a focal or global neurological deficit > 24 h; OR < 24 h if available neuroimaging documents a new haemorrhage or infarct; OR the neurological deficit results in death*
III. *TIA: duration of a focal or global neurological deficit < 24 h, any variable neuroimaging does not demonstrate a new hemorrhage or infarct*
IV. *No other readily identifiable non-stroke cause for the clinical presentation (e.g. brain tumour, trauma, infection, hypoglycemia, peripheral lesion, pharmacological influences), to be determined by or in conjunction with the designated neurologist. (Patients with non-focal global encephalopathy will not be reported as a stroke without unequivocal evidence of cerebral infarction-based upon neuroimaging studies)*
V. *Confirmation of the diagnosis by at least one of the following:*
    a. *Neurologist or neurosurgical specialist*
    b. *Neuroimaging procedure (CT scan or brain MRI), but stroke may be diagnosed on clinical grounds alone*

*Stroke classification*

I. *Ischemic: an acute episode of focal cerebral, spinal, or retinal dysfunction caused by infarction of the central nervous system tissue*
II. *Hemorrhagic: an acute episode of focal or global cerebral or spinal dysfunction caused by intraparenchymal, intraventricular, or subarachnoid hemorrhage*
III. *A stroke may be classified as undetermined if there is insufficient information to allow categorization as ischemic or haemorrhagic*

## Bleeding definitions:

*Bleeding will be defined according to different bleeding classifications (BARC, TIMI, PLATO, GUSTO). Bleeding information will be obtained in a manner which will allow the research team to classify each bleeding into different classifications.*

***Bleeding Academic Research Consortium Definition for Bleeding (BARC)***

*Mehran et al. Circulation 2011 Jun 14;123(23):2736-47.doi: 10.1161/CIRCULATIONAHA.110.009449.*

- ***Type 0:*** *no bleeding*
- ***Type 1:*** *bleeding that is not actionable and does not cause the patient to seek unscheduled performance of studies, hospitalization, or treatment by a healthcare professional; may include episodes leading to self-discontinuation of medical therapy by the patient without consulting a healthcare professional*
- ***Type 2:*** *any overt, actionable sign of haemorrhage (e.g., more bleeding than would be expected for a clinical circumstance, including bleeding found by imaging alone) that does not fit the criteria for type 3, 4, or 5 but does meet at least one of the following criteria: (1) requiring nonsurgical, medical intervention by a healthcare professional, (2) leading to hospitalization or increased level of care, or (3) prompting evaluation*
- ***Type 3***
  - ***Type 3a***
    - *Overt bleeding plus haemoglobin drop of 3 to <5 g/dL (provided haemoglobin drop is related to bleed)*
    - *Any transfusion with overt bleeding*
  - ***Type 3b***
    - *Overt bleeding plus haemoglobin drop ≥5 g/dL* (provided haemoglobin drop is related to bleed)*
    - *Cardiac tamponade*
    - *Bleeding requiring surgical intervention for control (excluding dental/nasal/skin/haemorrhoid)*
    - *Bleeding requiring intravenous vasoactive agents*
  - ***Type 3c***
    - *Intracranial haemorrhage (does not include microbleeds or hemorrhagic transformation, does include intraspinal)*
    - *Subcategories confirmed by autopsy or imaging or lumbar puncture Intraocular bleed compromising vision*
- ***Type 4:*** *CABG-related bleeding*
  - *Perioperative intracranial bleeding within 48 h*
  - *Reoperation after closure of sternotomy for the purpose of controlling bleeding*
  - *Transfusion of ≥5 U whole blood or packed red blood cells within a 48-h period*
  - *Chest tube output ≥2L within a 24-h period*
- ***Type 5:*** *fatal bleeding*
  - ***Type 5a:*** *Probable fatal bleeding; no autopsy or imaging confirmation but clinically suspicious*
  - ***Type 5b:*** *Definite fatal bleeding; overt bleeding or autopsy or imaging confirmation*

***TIMI bleeding classification:***

*Mehran et al. Circulation 2011 Jun 14;123(23):2736-47.doi: 10.1161/CIRCULATIONAHA.110.009449.*

**Non-CABG Related Bleeding:**

**1. Major**

- Any intracranial bleeding (excluding microhemorrhages <10 mm evident only on gradient-echo MRI)
- Clinically overt signs of hemorrhage associated with a drop in hemoglobin of ≥5 g/dL or a ≥15% absolute decrease in haematocrit
- Fatal bleeding (bleeding that directly results in death within 7 d)

**2. Minor**

- Clinically overt (including imaging), resulting in hemoglobin drop of 3 to <5 g/dL or ≥10% decrease in haematocrit
- No observed blood loss: ≥4 g/dL decrease in the haemoglobin concentration or ≥12% decrease in haematocrit
- Any overt sign of hemorrhage that meets one of the following criteria and does not meet criteria for a major or minor bleeding event, as defined above

    - Requiring intervention (medical practitioner-guided medical or surgical treatment to stop or treat bleeding, including temporarily or permanently discontinuing or changing the dose of a medication or study drug)
    - Leading to or prolonging hospitalization
    - Prompting evaluation (leading to an unscheduled visit to a healthcare professional and diagnostic testing, either laboratory or imaging)

**3. Minimal**

- Any overt bleeding event that does not meet the criteria above
- Any clinically overt sign of haemorrhage (including imaging) associated with a <3 g/dL decrease in haemoglobin concentration or <9% decrease in haematocrit

**Bleeding in the Setting of CABG:**

- Fatal bleeding (bleeding that directly results in death)
- Perioperative intracranial bleeding
- Reoperation after closure of the sternotomy incision for the purpose of controlling bleeding
- Transfusion of ≥5 U PRBCs or whole blood within a 48-h period; cell saver transfusion will not be counted in calculations of blood products.
- Chest tube output >2 L within a 24-h period

***GUSTO Bleeding Criteria:***

*Mehran et al. Circulation 2011 Jun 14;123(23):2736-47.doi: 10.1161/CIRCULATIONAHA.110.009449.*

**1.** *Severe or Life-threatening:*

- *Intracerebral hemorrhage*

- *Resulting in substantial hemodynamic compromise requiring treatment*

**2.** *Moderate:*
- *Requiring blood transfusion but not resulting in hemodynamic compromise*

**3.** *Mild*
- *Bleeding that does not meet above criteria*

***PLATO bleeding:***

*Schulman et al. J Thromb Haemost. 3 (4): 692–4. DOI:10.1111/j.1538-7836.2005.01204.x*

- *Major-Life Threatening:*
  - *Fatal*
  - *Intracranial*
  - *Intropericardiac with tamponade*
  - *Hypovolemic shock*
  - *Severe hypotension*
  - *Hemoglobin drop > 5g/dL*
  - *4 unit transfusion*

- *Major:*
  - *Clinically significant disability (e.g. intraocular hemorrhage with blindness)*
  - *Hemoglobin drop or 3-5 g/dl*
  - *2-3 unit transfusion*

- *Minor*
  - *Any bleeding requiring an intervention but not meeting major criteria*

## Aspirin and/or $P2Y_{12}$ switch / stop / start:

*During follow-up, relevant discontinuations or cessations of aspirin and/or the $P2Y_{12}$-inhibitor (clopidogrel, ticagrelor or prasugrel) will be recorded. The date and reason for discontinuation or cessation will be noted.*
